# Relative recency influences object-in-context memory

**DOI:** 10.1016/j.bbr.2014.12.024

**Published:** 2015-03-15

**Authors:** Shu K.E. Tam, Charlotte Bonardi, Jasper Robinson

**Affiliations:** School of Psychology, University of Nottingham, University Park, Nottingham NG7 2RD, United Kingdom

**Keywords:** OIC, object-in-context, RR, relative recency, SOR, spontaneous object recognition, Associative learning, Discrimination, Object recognition, Pavlovian conditioning, Priming, Recognition memory

## Abstract

In two experiments rats received training on an object-in-context (OIC) task, in which they received preexposure to object *A* in context *x*, followed by exposure to object *B* in context *y*. In a subsequent test both *A* and *B* are presented in *either* context *x* or context *y*. Usually more exploration is seen of the object that has not previously been paired with the test context, an effect attributed to the ability to remember *where* an object was encountered. However, in the typical version of this task, object *A* has also been encountered less recently than object *B* at test. This is precisely the arrangement in tests of ‘relatively recency’ (RR), in which more remotely presented objects are explored more than objects experienced more recently. RR could contaminate performance on the OIC task, by enhancing the OIC effect when animals are tested in context *y*, and masking it when the test is in context *x*. This possibility was examined in two experiments, and evidence for superior performance in context *y* was obtained. The implications of this for theoretical interpretations of recognition memory and the procedures used to explore it are discussed.

## Highlights

• Results of object-in-context experiments can be influenced by relative recency.
• Data from two experiments presented supporting this suggestion.
• This may complicate interpretation of results of object-in-context experiments.
• Recommendations are made on how to address this.
• Results consistent with an associative account of recognition memory.

## 1 Introduction

**Fig. 1:**
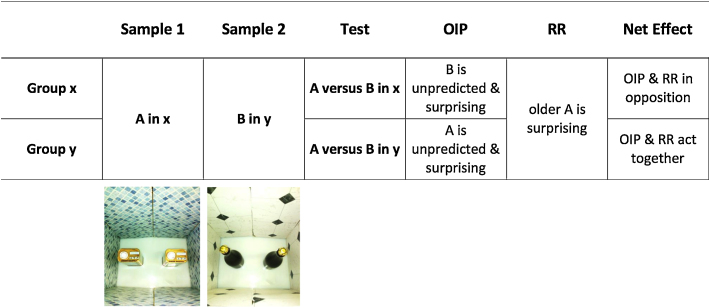
Design of Experiment 1 and example of specific objects and contexts presented in the sample phases of an OIC recognition trial. A rat is allowed to freely explore one type of object (*A*) in one visual context (*x*), and subsequently, a different type of object (*B*) in a different visual context (*y*). Following these two sample phases animals are given a choice between *A* and *B*, either in the less recent context x, or in the more recent context y. Note that object *A* has been encountered earlier than object *B*, and thus, at the time of test, the memory trace of object *A* would be relatively weaker than that of object *B*.

The phenomenon of spontaneous object recognition (SOR)—the observation that animals show a preference for exploring a novel object rather than one that has been previously encountered [1–3]—underlies a variety of tasks designed to examine memory processes in rats and mice. One version of this, the object-in-context (OIC) task, is used to assess rodents’ ability to remember *where* they have encountered a specific object. In one typical variant of this task the rodent is allowed first to freely explore one type of object, *A*, in one context, *x*, and subsequently a different type of object, *B*, in another context, *y* (see Fig. 1). Each context-object pairing usually consists of a single trial. After a delay, a test with objects *A* and *B* is given in one or other of the two contexts. It is typically reported that normal animals show a preference for the object that has *not* been encountered in the test context [4–12]. It has been proposed that this task relates to the ‘where’ component of episodic memory (e.g., [11,12]), context memory (e.g., [6]), recollection (e.g., [8]) or, more generally, contextual processing [7,9,10].

Another variant of the SOR procedure, the relative recency (RR) task, is designed to evaluate learning about *when* an object was experienced, by examining the animal's ability to discriminate objects based on how long ago they have been encountered. Here the rat is allowed to freely explore one type of object, *A*, and subsequently a different type of object, *B*, in the same apparatus. After a delay, the animal receives a test with the objects *A* and *B* presented simultaneously [13–18]. Animals show a preference for object *A*, the object that has been encountered earlier in the series. Relative recency has been described as a form of *temporal memory* in which events are remembered in sequence through a *higher-order mnemonic function* (e.g., [13,14]). This description implies that the mechanism underlying RR is likely to be quite distinct from that responsible for OIC learning—indeed the learning mechanisms involved in the OIC and RR versions of the task are regarded as independent [19].

The suggestion that RR and OIC tasks rely on independent mechanisms has, however, been challenged. For example, the most obvious analysis of SOR (e.g., [1–3]) is that exposure to object *A* leaves a memory trace that is present during the test, but absent for the new object, *B* [18,20–23]. Performance on RR tasks can be explained in similar terms, by arguing that the preference for the less recent *A* stems from its memory trace being weaker than that of the more recent *B*. Moreover, as the delay between the sample trials and testing increases, the difference in preference between *A* and *B* declines [18]. This may be understood by assuming that the difference in the trace strength of *A* and *B* is greatest at short delays when *A*'s memory trace has had an opportunity to decay but *B*'s remains active; as the delay increases, the memory traces for both objects will eventually decline to some negligible value (for discussion see, e.g., [23,24]). For ease of exposition, we describe the decline in memory performance over time as ‘*decay’* though we acknowledge here that it might be the result of a more active process of *interference* (see, e.g., [25]). That is, the memory trace of the target stimulus may be supplanted by the accumulated memory traces of interfering stimuli that are present during the retention interval. Interfering stimuli may be explicitly added by the experimenter (see, e.g., [25,26]) but even when they are not, non-specific events in the laboratory may create the same effect (see, e.g., [27]).

An alternative, but related, analysis of SOR maintains that apparatus cues enter into an excitatory association with the pre-exposed *A*—the process assumed to underlie Pavlovian conditioning. At test, the excitatory association activates the memory of the pre-exposed object, reducing the degree to which it is explored. In contrast the novel *B*, not being associated with the apparatus cues, is unexpected in that apparatus; thus its memory is not activated and normal exploration is maintained (e.g., [20,28–30]). The same explanation can be used to explain OIC learning: Although both objects will become associated with their respective contexts, only the memory of the object that has been previously encountered in the test context will be subject to this associative activation, and this is why it is explored less than the alternative object.

This view assumes that while RR depends on differential decay of the memory trace (cf. [15–17]), OIC stems from differences in associative activation of the memory trace [18,20–23,28–30], and both processes may contribute to SOR performance. This analysis of performance on both OIC and RR tasks in terms of the same underlying mechanism raises the possibility that they might interact. For example, in the OIC procedure originally described by Dix and Aggleton [5], animals received two exposures to each of the objects, *A* and *B*, in their respective contexts in a double alternation procedure (*A*, *B*, *B*, *A*); however in most OIC reports animals received only one preexposure to each of *A* and *B* [4,6–8,11,12,31]. This arrangement is problematic, because it renders interpretation of performance ambiguous (cf. [7,12]). For example, consider the case where *A* is pre-exposed in context *x*, and then *B* in context *y* (Fig. 1). When recognition is tested in the first, less recent context, *x*, the presence of *x* should result in a preference for object *B* because it has never been presented in *x*. But on the basis of relative recency one would anticipate the opposite—a preference for object *A*, because it has been encountered earlier in the series. As a result, the two processes would counteract each other when the test is conducted in the first context. In contrast, when recognition is tested in the second, more recent context, *y*, the context would lead to a preference for object *A*, which is the same object that would be preferred on the basis of relative recency. Thus here *both processes* would lead to a preference for object *A*. This would predict that OIC recognition should appear stronger when the test is conducted in *y*—the second and more recent context—than in *x*.

The preceding analysis does not deny the reality of the OIC effect; but it does suggest that, especially in this one-sample-trial variant, OIC performance can in part be attributed to the mechanism underlying RR. Moreover, although this suggestion is consistent with the proposal that both RR and OIC can be explained in terms of the same underlying mechanism, it does not require it: Provided one accepts the existence of both OIC and RR effects, the possibility that both might operate in the same task remains. This in turn implies that deficits in OIC performance produced by neural manipulations are ambiguous (e.g., [6–12,31])—a neural manipulation that affects OIC performance may indeed be the result of a change in learning about an object's location, but it could also be the result of a reduced relative recency effect in the subgroup tested in context *y*.

These arguments rest on the prediction that in the task variant described above, OIC performance is superior in context *y* than in context *x*. We were unable to evaluate this prediction on the basis of published work [4,6–12,31]; accordingly in the present article we describe the findings from our own OIC experiments which, although performed for different scientific reasons, allow comparison of performance in contexts *x* and *y* at test.

## 2 Experiment 1

Two groups of rats were trained in this version of the OIC task, receiving a single preexposure of object *A* in context *x* followed by a single preexposure of object *B* in context *y*. There followed a test in which animals were presented with objects *A* and *B* simultaneously, with animals in Group x being tested in context *x*, and those in Group y being tested in context *y* (Fig. 1). As outlined above, we predicted that for Group x, RR and OIC processes should counteract each other, resulting in a weaker difference in exploration between *A* and *B* than in Group y, for whom RR and OIC should both tend to produce more exploration of *A* than of *B*.

### 2.1 Method

#### 2.1.1 Subjects

The subjects were 12 male, Lister hooded rats (*Rattus norvegicus*; Charles Rivers, UK), housed in pairs in acrylic cages held in an air-conditioned vivarium, which was illuminated between 0700 and 1900. The cages contained fresh woodchip bedding, and a large cardboard cylinder to provide environmental enrichment; water and food were freely available. All the rats had previously served in a Skinner-box experiment, but were naive to the apparatus and stimuli described here. The experiment was conducted in two replications (*n* = 8 and *n* = 4 respectively; equal numbers within each replication were assigned to Groups x and y). At the start of the study rats in Replication 1 were approximately 350 g, and those in Replication 2 approximately 500 g. Two of the four rats in Replication 2 received small intra-peritoneal injections of physiological saline before each of the trials described below, which (based on work from our laboratory) was known not to influence behaviour; one of these rats was allocated to each experimental group. The procedures reported here constituted part of two, larger-scale experiments which included treatment groups that are not relevant to our current concerns and whose data are thus not considered here.

#### 2.1.2 Apparatus and stimuli

Four rectangular arenas with walls and floors of white translucent plastic (length × width × height: 60 cm × 40 cm × 45 cm), located in a quiet, brightly lit room, were used simultaneously. A camera was suspended 90.0 cm above the centre of each arena from a frame. Each camera's view (approximately 45° arc) included the complete floor but only the lower part of the four walls. Each camera was flanked by two LED spotlights, 22 cm apart, which produced a floor-level illumination of 50 lx; these were switched on at the start of each phase and off when the phase ended. Any-maze software (version 4.5; Stoelting, Wood Dale, IL) was used to track each rat's head location in two-dimensional space in the plane of the apparatus floor. The software was calibrated to detect a black region-of-interest (i.e., each rat's head) against a white background. The software created two notional circular *object zones* of 15-cm diameter, which surrounded each object's base; these zones were located along the horizontal midline of the apparatus, and the short walls were tangents to the zones; the centres of the circular zones were 30 cm apart. Rats’ head location in the two zones was timed continuously, which allowed the creation of a cumulative record of zone entry.

Multiple copies of three objects served as stimuli: (a) a blue, plastic bottle (20.0 cm in height with a circular base of 7.0 cm diameter); (b) a moulded plastic bottle covered with black and white penguins (20.5 cm in height with maximum base dimensions of 9.0 cm × 10.0 cm); and (c), a cylindrical, silver aluminium vacuum flask (19.5 cm in height with a base of 7.0 cm diameter).

Wall inserts made from medium density fibreboard lined with linoleum served as the contexts; these covered the whole of one of the shorter walls and half of both longer walls (see photographs in Fig. 1). These inserts were 45.0 cm high and, when inside the box, gave floor space of 42.0 cm × 32.0 cm. Two different patterns were used: *Mb*, a mosaic of 2.3 cm^2^ blue squares whose edges were 45° from horizontal (left-hand side of Fig. 1 photograph), and *Dw*, a mosaic of white 272-cm^2^ squares whose edges were 90° from horizontal, with a black, 16-cm^2^ square superimposed at each point where four white squares met horizontal (right-hand side of Fig. 1 photograph).

#### 2.1.3 Procedure

##### 2.1.3.1 Apparatus pre-exposure

Each rat was exposed to the arena (without objects or inserts) for three sessions, one session per day. In each session the rat was placed in the apparatus facing the midpoint of the long wall and allowed to explore for 10 min. The wall inserts and the floor of the apparatus were cleaned with diluted alcohol after each rat's session.

##### 2.1.3.2 Sample trials

Each rat received two sample trials of 10-min duration. In the first the rat was allowed to explore two copies of a novel object (*A*) in one context: the two copies of *A* were placed at the centres of the two object zones. Five minutes later, rats received the second sample phase, in which they were allowed to explore two copies of a different novel object (*B*), positioned in a similar manner in the alternative context. In both replications, half the rats in each group had context *x* as *Mb* and context *y* as *Dw*, and the remainder the reverse; in Replication 1 half of each of these two subgroups had the blue bottle as *A* and the penguin as *B*, and the remainder the reverse; in Replication 2 objects *A* and *B* were the penguin and either a steel flask (*n* = 3) or the blue bottle (*n* = 1).

##### 2.1.3.3 Test trials

The test trial occurred approximately 5 min after the final sample trial. Rats in Group x were tested in context *x* (the first-sampled context) and rats in Group y in context *y*. These trials were identical to the sample trials except that animals were presented simultaneously with one copy of *A* and one of *B*. For approximately half the rats in each group, *A* was on the left and *B* on the right, and for the remainder the reverse. Data were recorded for the first two minutes of the test.

#### 2.1.4 Data treatment

No data were recorded during the apparatus preexposure or sample trials. OIC recognition performance during the test was expressed as the ratio (*n* − *p*)/(*n* + *p*), where *p* and *n* are the times spent exploring the object that, respectively, had and had *not* been paired with the test context. This measure yields ratios ranging between +1 and −1, with +1 representing exclusive exploration of the object that had not been presented in that context during its sample trial (the expected OIC result) and 0 representing equivalent exploration of the two objects. Ratios were calculated for each of the first two minutes of the test.

### 2.2 Results

**Fig. 2:**
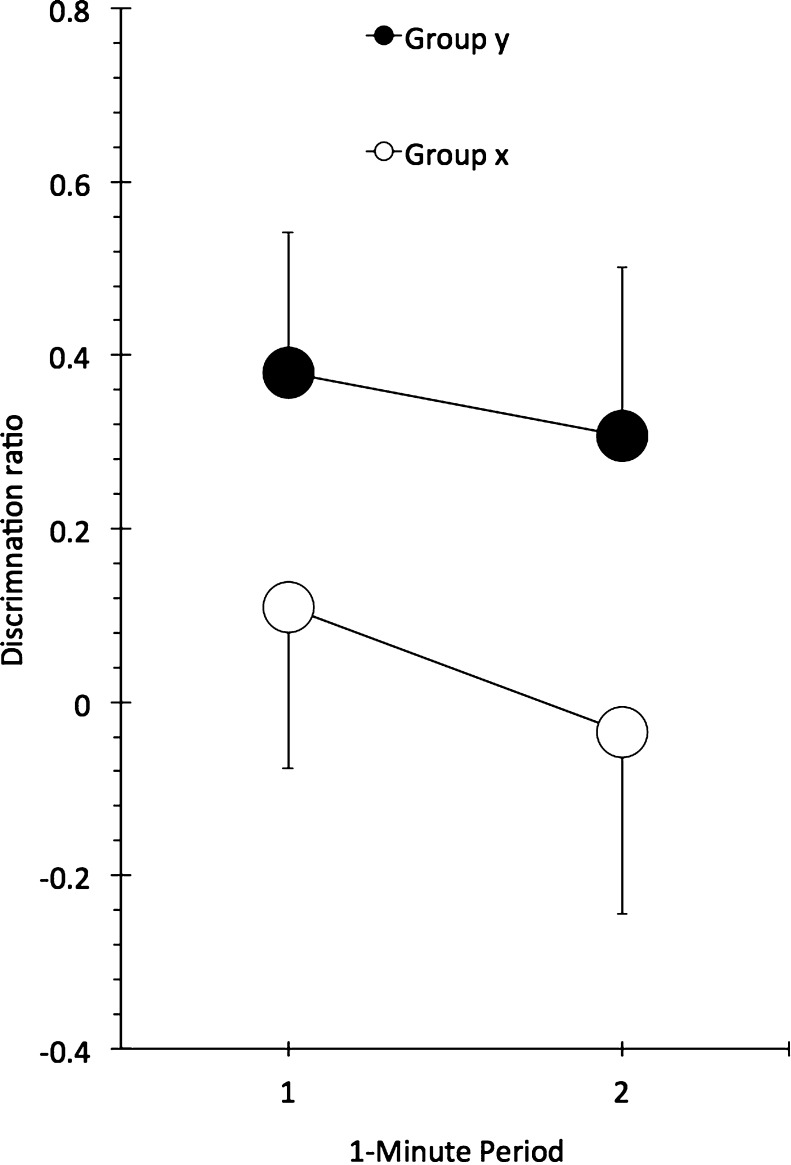
Mean discrimination ratios for the first and second minutes of the test of Experiment 1. Bars show standard error of the mean.

The test data are summarized in Fig. 2, and indicate a stronger OIC bias in Group y than in Group x. An analysis of variance (ANOVA) with group and minute as factors confirmed this description, yielding a reliable main effect of group only, *F*(1, 10) = 7.7, *p* < .020, *MSE* = .073, $\eta_{p}^{2}=.436\text{,}$ 95% CI = [.01, .68]; neither the main effect of minute nor the interaction was reliable, smallest *p* > .100, *F*(1, 10) = 3.2, *MSE* = .022.

### 2.3 Discussion

Our prediction was that performance would be better when the animals were tested in context *y* than context *x*, because in the former case RR facilitates performance while in the latter it opposes it—and that is exactly what we observed. In contrast, Norman and Eacott [7] examined performance separately in contexts *x* and *y*, but reported that this factor had no statistical impact on their results. It is not clear what could have produced this discrepancy, although Norman and Eacott's study differed in a number of ways from Experiment 1. For example, in their experiment the rats’ exploration of the objects was limited to 30 s in each sample phase. This could have reduced the strength of the objects’ memory traces at test, thus reducing the contribution of RR to test performance. Mumby et al. [6] also report a non-significant influence of testing in contexts *x* and *y*, although their results numerically paralleled those of our Experiment 1, showing superior performance in context *y* than in context *x*.

There are two other potential explanations for the results of Experiment 1 that must, however, be considered before we accept that RR may occur in OIC tasks. First, the general experimental environment may have been less familiar to the rats during the first sample trial in *x* than during the second sample phase in *y*. Thus, through processes seen in standard object recognition (e.g., [1,2]), these differences in context familiarity might have resulted in more exploration of *x* than of *y*. Such a bias could in turn influence exploration of the objects, resulting in less exploration of object *A* than of object *B*. A tendency for the first-presented object to be less well processed, rather than the fact that it was less recent, could have been the reason why performance was worse in Group x than in Group y. The second possibility is that both objects were processed equally well in the two sample trials, but that when animals were tested in context *x*, because it had been experienced longer ago than context *y*, it might have elicited more competing responses, which masked OIC performance.

**Fig. 3:**
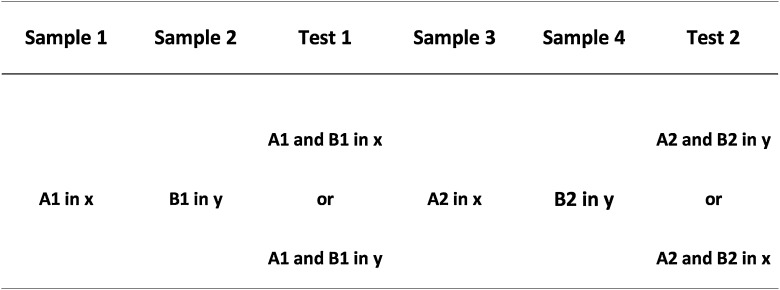
Design of Experiment 2.

Both these alternative explanations rely on the suggestion that the two contexts differed in their familiarity, either during preexposure or at test. Such a difference is an intrinsic part of the procedure and so cannot be entirely eliminated, but it can be minimized. This was the purpose of Experiment 2 (Fig. 3). In this study all rats were preexposed to both contexts before the experiment began, and the experiment was conducted within subjects, so that all rats received training with two sets of objects in the two contexts, but were tested with one set of objects in context *x*, and with the other in context *y*. Both these factors would increase the familiarity of both contexts and encourage full habituation of potential competing exploratory behaviour. In addition, the interval between the second sample and the test phase was extended from 5 min to 2 h, thus minimizing differences in the relative recency of the two contexts. Finally, we recorded exploration during the two sample trials, to explicitly evaluate the possibility that this could have differentially biased exploration of the objects during the preexposure phase.

## 3 Experiment 2

### 3.1 Method

#### 3.1.1 Subjects

Fifteen Lister Hooded male rats (Harlan, Bicester, UK) were used in the present study. They had all received sham operations of the hippocampus; thus they were anaesthetized, scalp incized, facial muscles retracted and a portion of the cranial bone above the hippocampus removed with an electric drill. A 25-G, bevel-tip needle was lowered to a series of sites in the hippocampus, but no neurotoxin was passed. Then the scalp was sutured, and they were allowed to recover for two weeks before the start of behavioural testing (full details of procedure are described in [18]), at which time they weighed approximately 400 g. They were caged in pairs exactly as in Experiment 1. Before the start of this study they were tested on different versions of the SOR task, but were naive with respect to both the contexts and the objects used in the present study.

#### 3.1.2 Apparatus and stimuli

Most of the apparatus was identical to that employed in Experiment 1. Different objects were used on each recognition trial; they were made of various materials (e.g., plastic, glass, stainless steel, and ceramic) and were of different sizes and shapes. There were two pairs of objects in total, and multiple copies of each type of object. The pair of objects used in the first cycle comprised a ceramic model duck (15 cm long and 7 cm wide and 9 cm in height) and a filled, shaped glass bottle (with a base diameter of 7 m, and 9 cm high). The pair of objects used in the second cycle comprised a rectangular olive oil can (with a rectangular base 10 cm × 7 cm and 17.5 cm high), and a black glass bottle (with a base 9 cm in diameter and 32 cm high). The zones round the objects in the present experiment were 10 cm in radius, but otherwise arranged as in the previous experiment.

#### 3.1.3 Procedure

##### 3.1.3.1 Apparatus pre-exposure

Each rat was exposed to the arena *without* any objects for four days, for two sessions per day. In one of these two 10-min sessions, the wall inserts *Mb* were put in the arena, while for the other the wall inserts *Dw* were used. In all other respects these sessions were identical to those of Experiment 1.

All rats received two cycles of the following OIC procedure, one on each of two consecutive days. Each cycle used a different pair of objects, but the same contexts.

##### 3.1.3.2 Sample and test trials

The sample trials were identical to those of Experiment 1, except that each sample phase lasted only 5 min. The test trials took place two hours after the end of the second sample phase, but were otherwise similar to those of Experiment 1. For the first OIC sequence, 8 rats were tested in context *x* and 7 rats in context *y*; for the second all rats that were first tested in context *x* were now tested in context y, and vice versa. For the first OIC sequence half of each of these groups (*ns* = 4 and 3) had *Mb* as the context insert in the first sample phase and *Dw* in the second sample phase; this arrangement was reversed for the remaining rats. Half of each of these subgroups (*n*s = 2 and 1) had the duck in the first sample phase and the bottle in the second, and for the remainder the reverse. Approximately half of each of these subgroups were tested with duck on the left and bottle on the right, and for the remainder the reverse. In the second OIC sequence the animals that had received *Mb* as the context insert in the first sample phase and *Dw* in the second sample phase continued to do so, as did the animals that had experienced the reverse. The counterbalancing of the two new objects used in this phase, the can and black bottle, was identical to that in the first sequence.

### 3.2 Results

One aim of this experiment was to examine whether there was less total exploration during the first than the second sample trial; accordingly exploration during the two sample trials from each of the two test cycles was computed. Initial inspection revealed no influence of the test sequence (test in *x* before test in *y* or vice versa), and so data were collapsed across this factor. The mean exploration time for the first and second sample trials (with SEM in parentheses) were 157.5 s (1.3 s) and 151.2 s (1.2 s) for cycle 1, and 152.0 s (1.6 s) and 160.0 s (1.5 s) for cycle 2. An ANOVA with cycle (first and second) and sample trial (first and second) as factors revealed no reliable main effects, *F*s < 1, or interaction between those variables, *F*(1, 14) = 1.4, *p* > .263, *MSE* = 562.337. Thus there was no evidence for greater competing responses in the first sample trial, and thus no reason to suppose that differential exploration of the two contexts had a systematic effect on exploration of the two objects.

**Fig. 4:**
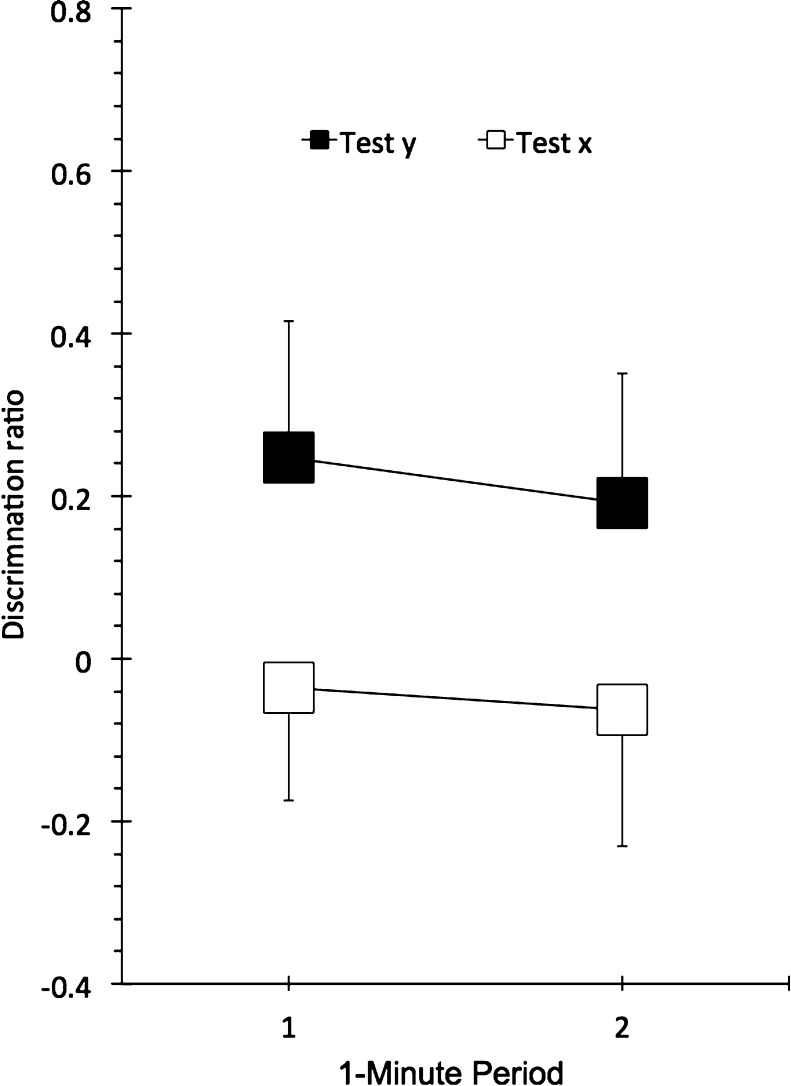
Mean discrimination ratios for the first and second minutes of the test of Experiment 2. Bars show standard error of the mean.

The test data are summarized in Fig. 4. A test context (*x* versus *y*) × minute of test (1st and 2nd) repeated-measures ANOVA revealed a main effect of test context, *F*(1, 14) = 4.6, *p* < .050, *MSE* = .168, $\eta_{p}^{2}=.250$, 95% CI = [.00, .93], again showing OIC performance to be better in context y than in context *x*. The main effect of minute of test and the test context × minute of test interaction were not reliable reliable, *p*s > .40.

### 3.3 Discussion

The results of this experiment confirm those of Experiment 1, in showing a greater OIC effect when animals were tested in the more recent context. Moreover, some potential alternative explanations of the results of Experiment 1 were addressed. First, there was no evidence of any difference in exploration in the two sample phases in either cycle of testing, making it difficult to argue that object *A* was preferred not because it was less recent per se, but because it had been less effectively explored than object *B*, as a result of competing behaviour provoked by the relatively novel environment on the first sample trial. Moreover, the increased experience with the two contexts, and the increased delay between the second sample phase and the test, will have minimized the possibility that context *x* selectively interfered with performance on test simply because it had been experienced less recently. Thus the results of these two experiments are consistent with our prediction that RR can influence performance in OIC tasks.

## 4 General discussion

The present results demonstrate that in the most commonly used version of the OIC task (e.g., [4,6–12,31]), in which animals receive only one sample trial with each of the two objects, performance was reliably better when the animals were tested in the most recent context. We have interpreted this as evidence that performance does not depend solely on the context in which the test is conducted, but also on the RR of the two test objects [16–18,20]—The suggestion is that, when animals are tested in the first of the two contexts, RR opposes the effect of the context, whereas when they are tested in the second it exaggerates it. This version of the task does not, therefore, provide a pure measure of the ability of animals to encode the context in which a particular object was presented, or the effect of this on subsequent memory performance.

### 4.1 Empirical implications

We offer four recommendations to minimize the impact of RR and/or to examine the size of RR in OIC experiments:

#### 4.1.1 Length of retention interval

According to the analysis we have offered [18,20,28–30], extension of the interval between the final sample trial and testing (the retention interval) may allow the memory traces from the sample trials to decay to their inactive states (i.e. eliminating the source of RR). With such an arrangement, only the intended, retrieval-based source of test performance will occur on test (i.e. OIC learning). The point at which decay is complete could be determined by systematic variation of the retention interval in RR experiments: In particular, the point at which the retention interval eliminates RR discrimination will correspond to the point at which the sample memories have become inactive. The effective retention interval can then be used to accurately assess OIC learning. In the absence of such information it would seem sufficient to use 24-h retention intervals [9,10], which are likely to be sufficiently long to allow complete memory decay.

#### 4.1.2 Number of preexposure trials

Some modifications to the design of the OIC experiment can be used to reduce RR. A more complex version of the OIC procedure, originally employed by Dix and Aggleton [5], is less susceptible to the potential influence of RR, because here the two objects are preexposed twice in a double alternating sequence (i.e., *xA*, *yB*, *yB*, *xA* for half of the rats and *yB*, *xA*, *xA*, *yB* for the remainder). This treatment ensures that the presentation order of the two objects is matched (i.e., object *A* being pre-exposed in positions 1 and 4, and *B* in positions 2 and 3, each with an identical midpoint). Of course whether this matching in terms of presentation order can be translated directly into matching of memory traces is questionable, as we cannot safely assume that the memory trace from an object exposed on the first and fourth trials is equivalent to that presented on the second and third. Such inequality may be the result of a nonlinear decay rate—a characteristic of memory performance (e.g., [32–34])—or from differential interference stemming from differences in the number of intervening preexposure trials [27]. Thus even the use of double-alternating sequences does not entirely avoid potential effects of RR. It is also notable that some researchers may wish to use a pair of single sample trials to model episodic memory processes [16,31,35], although some have questioned the logic of mapping single-trial learning onto episodic memory [36].

#### 4.1.3 Arrangement of preexposure trials

A third variant of the OIC procedure avoids the potential influence of RR by combining the two sample pairings into a single trial. For example, Good, Barnes, Staal, McGregor, and Honey [16] gave rats a single sample trial in which four objects were located in the four corners of the experimental arena. In the subsequent test, the locations of two of the objects that were located in opposite corners were switched. We may assume that the switched objects may have entered into association with the two adjacent, un-switched objects during the single sample trial or with extra-arena cues in the laboratory. These associations can be thought of as analogues of the *x/y* context associations in other OIC procedures; thus comparison of exploration of the switched and unswitched objects gives a measure of OIC learning.

#### 4.1.4 Analysis of subgroups’ data

Even when used with the recommendations above, our results demonstrate the importance of analysis of data from the counterbalanced subgroups that receive testing in context *x* or context *y*. The outcome of this analysis may be that there is no evidence of the involvement of RR in OIC (cf. [6,7]), in which case results such as the effects of neural manipulations may be interpreted straightforwardly. However, when RR is detected during OIC, effects of neural manipulations are ambiguous—Their sources could be, e.g., a deficit in RR, OIC, or both. For the same reasons it may also be interesting for researchers to report such new analyses of their previously published work.

### 4.2 Theoretical interpretation

We noted above that it is possible to explain both RR and OIC performance in terms of a mechanism that compares the relative strength of the memory traces of the two test objects. According to this view, RR depends on differential decay of the memory trace (cf. [15–17]), while OIC results from differences in the degree to which it is associatively activated [18,20–23,28–30]. These assumptions have been formally incorporated into an influential account of associative learning termed SOP (Sometimes Opponent Process) [21,22,37,38], and this SOP model has been used successfully by some authors to explain performance in object memory tasks [18,20,28–30]—representing a challenge to the views that performance in recognition tasks of the type described here is independent of associative learning processes [39], or that it is the result of more complex processes [5–14 for a recent review see 40]. According to SOP, the presentation of stimuli (objects or the context in which they are presented) is assumed to elicit activity in a population of representational elements, coding for identity of each stimulus. Initially, elements are in a primary state of activity, which elicits relatively high levels of responding (e.g., approach) and which supports the formation of excitatory associations. Elements next pass into a secondary state of activation that does not allow excitatory associative learning, and supports weaker approach responding. Ultimately the elements become inactive. A crucial assumption of this model is that elements cannot pass directly from their secondary to their primary activity states. This reduces approach responding when an object is presented again after recent presentation, because a relatively large proportion of its elements are still likely to be residing in their secondary states of activity. But more remotely presented objects, whose elements have entirely decayed to inactivity, will be able to elicit stronger approach responding because their elements can pass directly into their primary activity state. These assumptions provide an explanation of performance in an RR experiment: at test a greater proportion of the *less* recent object's elements will have become inactive, allowing them to enter the primary activation state when the object is presented at test [18,28]. Similar logic can offer an explanation of performance in the simple SOR task, as the novel object's elements are *all* able to enter the primary activation state, whereas those of the familiar object are not.

A second critical assumption of SOP is that when two items are associated, presentation of one will place elements of the other directly into the secondary activation state. This provides an alternative analysis of SOR, by supposing that apparatus cues enter into an excitatory association with the object during the pre-exposure phase. At test, the apparatus cues place the elements of the pre-exposed object into the secondary activation state, reducing the degree to which it is explored. In contrast the novel object, being un-associated with the apparatus cues, is not expected in the apparatus, so its elements are inactive and can enter the primary activation state when it is presented; thus normal levels of exploration are maintained [18,20,28–30]. OIC performance can be explained in a similar manner; although both objects are associated with their respective contexts, at test only the elements of the object in its preexposure context will be associatively activated. Thus the elements of the alternative object will be more able to enter the primary activation state and elicit the exploration response.

Thus SOP proposes that RR depends on differential *decay* of the stimulus elements of the two objects, while OIC stems from differences in their *associative activation*—and there is nothing in the model which would preclude both processes operating in the same task. Thus a model of this type should easily be able to accommodate the results reported here—a suggestion which we have confirmed in unpublished computer simulations. Moreover, although it can explain performance on these tasks as well as other theories of object recognition, it can also explain a great variety of associative learning effects. This breadth, we would argue, gives this model an edge over those whose explanatory power is limited to object recognition memory.

## 5 Conclusion

The results of the present experiments demonstrate that, in the most widely used variant of the object-in-place task, relative recency can contribute to performance. This possibility potentially complicates interpretation of the results from experiments using this procedure, and we suggest a number of recommendations to minimize, or at least monitor, the effect of RR on OIC performance. Our results are also consistent with an associative account of recognition memory, which explains performance in SOR, RR and OIC tasks in terms of the same underlying mechanism.
